# Imaging in Lyme neuroborreliosis

**DOI:** 10.1007/s13244-018-0646-x

**Published:** 2018-09-04

**Authors:** Elisabeth S. Lindland, Anne Marit Solheim, Silje Andreassen, Else Quist-Paulsen, Randi Eikeland, Unn Ljøstad, Åse Mygland, Ahmed Elsais, Gro O. Nygaard, Åslaug R. Lorentzen, Hanne F. Harbo, Mona K. Beyer

**Affiliations:** 1 Department of Radiology and Nuclear Medicine, Oslo University Hospital, Sognsvannsveien 20, 0372 Oslo, Norway; 2 Department of Radiology, Sorlandet Hospital, Sykehusveien 1, N-4809 Arendal, Norway; 3 Institute of Clinical Medicine, University of Oslo, Oslo, Norway; 4 Department of Neurology, Sorlandet Hospital, Kristiansand, Norway; 5 Institute of Clinical Medicine, University of Bergen, Bergen, Norway; 6 Addiction Unit, Sorlandet Hospital, Arendal, Norway; 7 Department of Infectious Diseases, Oslo University Hospital, Ullevaal, Norway; 8 The Norwegian National Advisory Unit on Tick-Borne Diseases, Sorlandet Hospital, Arendal, Norway; 9 Department of Neurology, Oslo University Hospital, Oslo, Norway; 10 Department of Life Sciences and Health, Oslo and Akershus University College of Applied Sciences, Oslo, Norway

**Keywords:** Lyme neuroborreliosis, MRI, Neuritis, Myelitis, Encephalitis

## Abstract

**Abstract:**

Lyme neuroborreliosis (LNB) is a tick-borne spirochetal infection with a broad spectrum of imaging pathology. For individuals who live in or have travelled to areas where ticks reside, LNB should be considered among differential diagnoses when clinical manifestations from the nervous system occur. Radiculitis, meningitis and facial palsy are commonly encountered, while peripheral neuropathy, myelitis, meningoencephalitis and cerebral vasculitis are rarer manifestations of LNB. Cerebrospinal fluid (CSF) analysis and serology are key investigations in patient workup. The primary role of imaging is to rule out other reasons for the neurological symptoms. It is therefore important to know the diversity of possible imaging findings from the infection itself. There may be no imaging abnormality, or findings suggestive of neuritis, meningitis, myelitis, encephalitis or vasculitis. White matter lesions are not a prominent feature of LNB. Insight into LNB clinical presentation, laboratory test methods and spectrum of imaging pathology will aid in the multidisciplinary interaction that often is imperative to achieve an efficient patient workup and arrive at a correct diagnosis. This article can educate those engaged in imaging of the nervous system and serve as a comprehensive tool in clinical cases.

**Key Points:**

• *Diagnostic criteria for LNB emphasise exclusion of an alternative cause to the clinical symptoms.*

• *MRI makes a crucial contribution in the diagnosis and follow-up of LNB.*

• *MRI may have normal findings, or show neuritis, meningitis, myelitis, encephalitis or vasculitis.*

• *White matter lesions are not a prominent feature of LNB.*

## Introduction

Lyme borreliosis is an infection with the spirochete *Borrelia burgdorferi (Bb)*. It is transmitted through tick bites, and is the most common vector-borne disease in Europe and North America [1, 2]. Estimates of prevalence are 100-130 per 100,000 in Europe, and 20-100 cases per 100,000 people in the United States (US) [3]. Involvement of the nervous system is reported to occur in 10-15% of patients with borreliosis [4].

In Europe, the dominant strains are *Borrelia garinii* and *Borrelia afzelii*, whereas *Borrelia burgdorferi* sensu stricto is the only pathogen in American Lyme borreliosis [4–6].

Lyme neuroborreliosis (LNB) is an important differential diagnosis in patients with nonspecific nervous system MRI findings and possibility of previous exposure to tick bite. In this article, we explain the diagnostic criteria, summarise and discuss current knowledge of imaging and pathology in LNB and describe the imaging findings in a spectrum from the typical presentations (meningoradiculitis, cranial nerve involvement), to less common (myelitis, peripheral neuropathy) and rare disease courses (meningoencephalitis, vasculitis).

## Disease manifestations

The clinical presentation of LNB can vary widely, partly due to the genetic differences in spirochetal strains. Individual variations in immunological response and the possibility of co-infections may also explain the wide spectrum of symptoms that can be encountered. The skin is primarily affected, and erythema migrans can be observed. About one third to half of patients with LNB recall a tick bite or a rash [7, 8]. The most common neurological manifestation of LNB in adults is the Bannwarth syndrome with painful radiculoneuritis and aseptic meningitis, the former is most prominent in Europe and the latter in North America [1]. LNB with facial nerve palsy is commonly encountered in both continents. Rarer manifestations from the nervous system are meningoencephalitis, encephalomyelitis, cerebral vasculitis and peripheral neuropathy [6]. LNB may rarely present to the ophthalmologist with optic neuropathies and papilledema due to raised intracranial pressure [9]. The distinction between early and late LNB is made from duration of neurological symptoms of less or more than 6 months, and more than 95% of cases are classified as early disease [10]. Most LNB patients respond well to antibiotic treatment, but some experience persistent complaints [11, 12]. A definition for post-Lyme disease syndrome has been suggested to include subjective symptoms of fatigue, cognitive symptoms and/or widespread musculoskeletal pain starting within 6 months after completed treatment [13]. Post-Lyme disease syndrome is not discussed in this article.

## Diagnostic criteria

The European Federation of Neurological Societies (EFNS) established guidelines for LNB diagnosis in 2010 [10]. The criteria for LNB diagnosis are: LNB compatible neurological symptoms and signs with no other explanation, pleocytosis and production of *Bb* specific antibodies in the cerebrospinal fluid (CSF). Intrathecal antibody production is often expressed as a positive *Bb* antibody index (AI), where measurements of antibody show a higher level in CSF than in serum [14]. The European guidelines state that the condition is possible when two out of three criteria are present, and definite when all three criteria are met (Table 1). Direct test methods for detection of *Bb*, mainly culture and polymerase chain reaction, have low sensitivity and are seldom helpful in the diagnostic work-up [14]. Interpretation of serology can also be a challenge in the diagnostic process, since the sensitivity is low in the early stage of infection. Further, both IgM and IgG antibodies can remain positive several years after infection, so results may not differentiate previous exposure, reinfection or acute infection [14, 15]. American Academy of Neurology (AAN) diagnostic criteria do not include analysis of cerebrospinal fluid, but presence of one or more of erythema migrans, direct proof through histopathology, microbiology or polymerase chain reaction or immunologic evidence of infection [4]. To increase specificity of serology, a two-tier approach is recommended with an initial enzyme immunoassay followed by separate immunoglobulin Western blots if the first step is positive [14]. Both American and European diagnostic criteria for LNB emphasise the exclusion of other apparent cause to the clinical disorder [4]. Magnetic resonance imaging (MRI) therefore makes a crucial contribution in the diagnosis and follow-up of LNB.

**Table 1:** EFNS diagnostic criteria for LNB^a^ [10]

Criteria	Definite LNB	Possible LNB
1) Neurological symptoms suggestive of LNB without other obvious reasons 2) Cerebrospinal fluid pleocytosis^b^ 3) Intrathecal *Bb* antibody production	All three criteria fulfilled	Clinical criteria and one of two laboratory criteria fulfilled

*EFNS* European Federation of Neurological Societies, *LNB* Lyme neuroborreliosis

^a^Special criteria apply for late LNB with polyneuropathy [10]

^b^>5 white blood cells per mm^3^

## Pathology

A recent study with inoculation of *Bb* infection in the central nervous system (CNS) of rhesus macaques resulted in lymphocytic neuritis and ganglionitis, and neuronal degeneration and demyelination in the peripheral nervous system. In brainstem and medulla there were lesions with focal malacia and nerve fibre degeneration, and some subjects had myelitis with necrosis and degeneration [16]. The experiment gave evidence that the nerve injuries are mainly due to immune mediated mechanisms. Human studies are limited, but have pointed mainly to axonal damage in LNB mononeuropathy and plexopathy [4, 17]. The myelitis seen in most LNB cases could be the inflammation of radiculoneuritis extending to the spinal cord, CNS parenchymal involvement is otherwise considered rare in LNB [4]. *Bb* invasion into the human brain has been reported in four cases with biopsy from MRI enhancing lesions [18–20].

## Imaging in Lyme neuroborreliosis

Early imaging reports, starting with Halperin et al. in 1988 and 1989, focused on non-specific white matter changes in Lyme encephalopathy, considered a late phase of LNB with mild cognitive deficits [21, 22]. These and six other studies published between 1990 and 2007 included 10-27 subjects, and white matter lesions were found in 15-63% of the patients [23–28]. The diagnostic criteria in these reports varied, and for many cases would not fulfil the EFNS or AAN criteria of today. Sequence development and higher resolution of MRI have constantly evolved with increased sensitivity of this imaging method, and knowledge of age-related white matter changes has also changed throughout this time. Therefore, the claim that LNB causes non-specific white matter changes should be considered based on weak scientific evidence. In 2009 Agarwal and Sze reported imaging data in 66 patients with LNB. Seven patients, but also six out of the 50 healthy control subjects, had white matter lesions, and the result suggests that white matter lesions are not a feature of LNB. One patient had an enhancing parenchymal lesion and three had meningeal or cranial nerve enhancement [18]. Numerous case reports demonstrate that LNB has a wide spectrum of imaging entities, including diffuse or tumour-like affection in brain or spinal cord [19, 20, 29–34], meningeal and/or nerve enhancement [33–43], as well as vascular affection with stroke-like presentation [44–54] or haemorrhage [35, 55, 56]. Overlap of imaging findings between LNB and multiple sclerosis (MS) has been discussed [1, 57], but is understudied.

There is limited knowledge of structural and functional changes in LNB with quantitative MRI techniques. Three studies have reported structural changes, and no difference between the patients and controls was found [23, 27, 28]. However, these studies included a limited number of subjects and controls (*n* = 6-20), so they probably lacked power to show subtle changes. Functional studies have been conducted with PET [58, 59] and with SPECT [26, 60–62]. These studies reported regional hypoactivity and hypoperfusion, but were hampered by heterogeneous study populations with non-specific symptoms and included individuals with uncertain diagnoses. Larger, prospectively conducted case-control studies are needed to learn more about imaging abnormalities in LNB.

## Meningoradiculitis

**Fig. 1:**
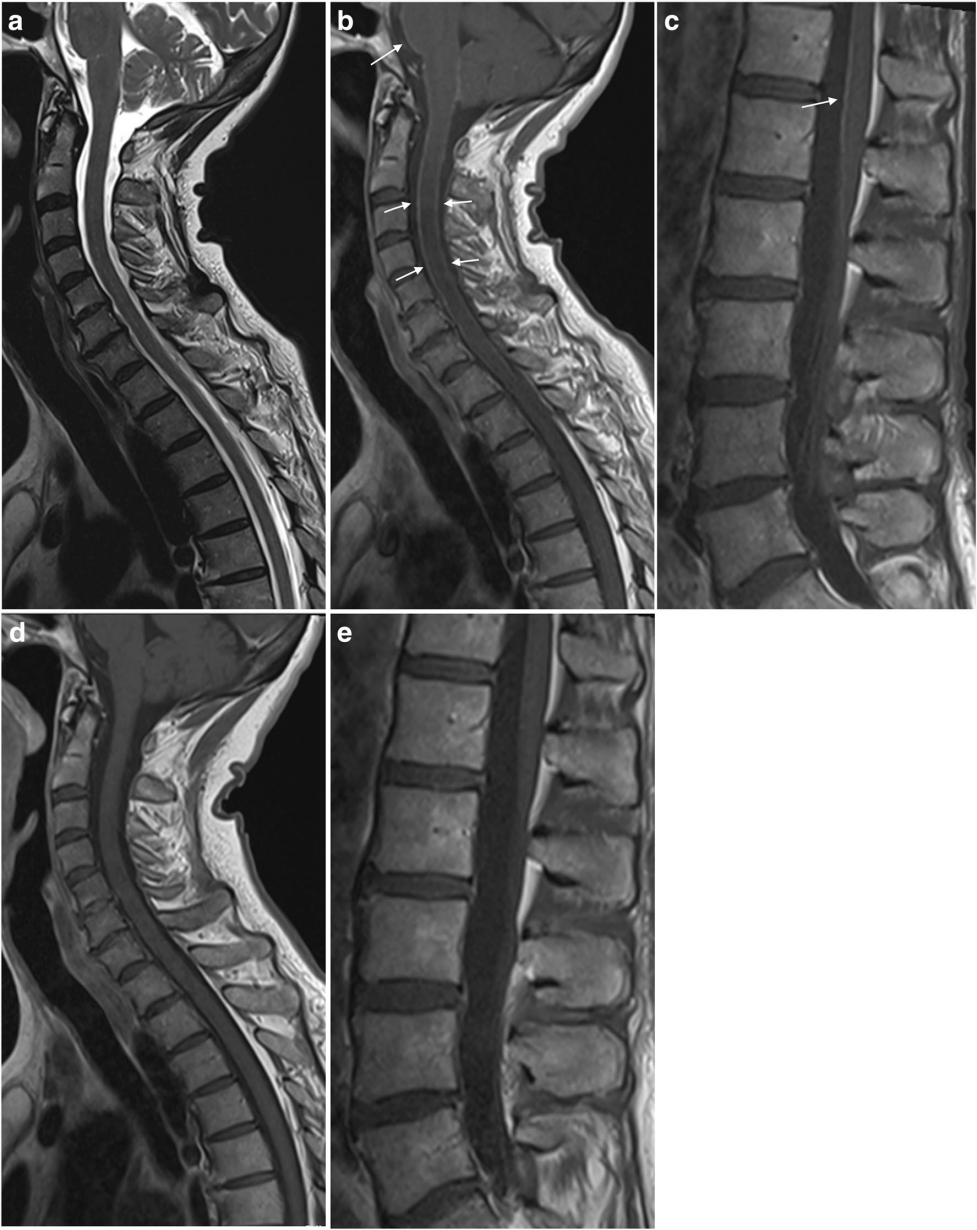
Meningoradiculitis due to Lyme neuroborreliosis (LNB) in a 73-year-old female. She experienced periods of strong superficial abdominal pain, reduced appetite and headache. Sagittal T2 (**a**) and T1 after gadolinium-based contrast agent injection (**b**, **c**) demonstrated normal cord parenchyma and diffuse leptomeningeal enhancement (arrows) of spinal cord and brain stem. Cerebrospinal fluid (CSF) analysis showed pleocytosis and intrathecal production of *Borrelia burgdorferi* (*Bb)*-specific IgM and IgG antibodies. No leptomeningeal enhancement was found 4 weeks after the treatment on post-contrast sagittal T1 weighted images (**d**, **e**)

The most typical manifestation of nervous system involvement in European Lyme borreliosis is the Bannwarth syndrome with radiculoneuritis, causing radicular pain and sometimes paresis of extremities or the abdominal wall [1, 7]. Patients often describe nocturnal exacerbation of the pain, and location and intensity may vary [10]. Studies report 19.5 – 29% of LNB cases to have clinical signs of meningitis [7, 8]. Enhancement of meninges, cranial and spinal nerves can be encountered. Images in Fig. 1 show a case of LNB with meningoradiculitis where leptomeningeal enhancement was diffuse and widespread, and disappeared promptly with treatment. The frequency of leptomeningeal enhancement on MRI examination in LNB has only been reported in the above mentioned retrospective study by Agarwal and Sze. One of 63 LNB cases with contrast enhanced MRI examination in that study showed mild leptomeningeal enhancement [18]. For bacterial meningitis in general, post-contrast T1-weighted images yield a low rate of leptomeningeal enhancement, while the use of post-contrast FLAIR sequence increases sensitivity of MRI [63, 64]. It is also important to be aware of the rather low specificity of meningeal enhancement with T1-weighted images, mainly due to interpretation disturbed by the normal enhancement in vascular structures [65].

## Cranial neuritis

**Fig. 2:**
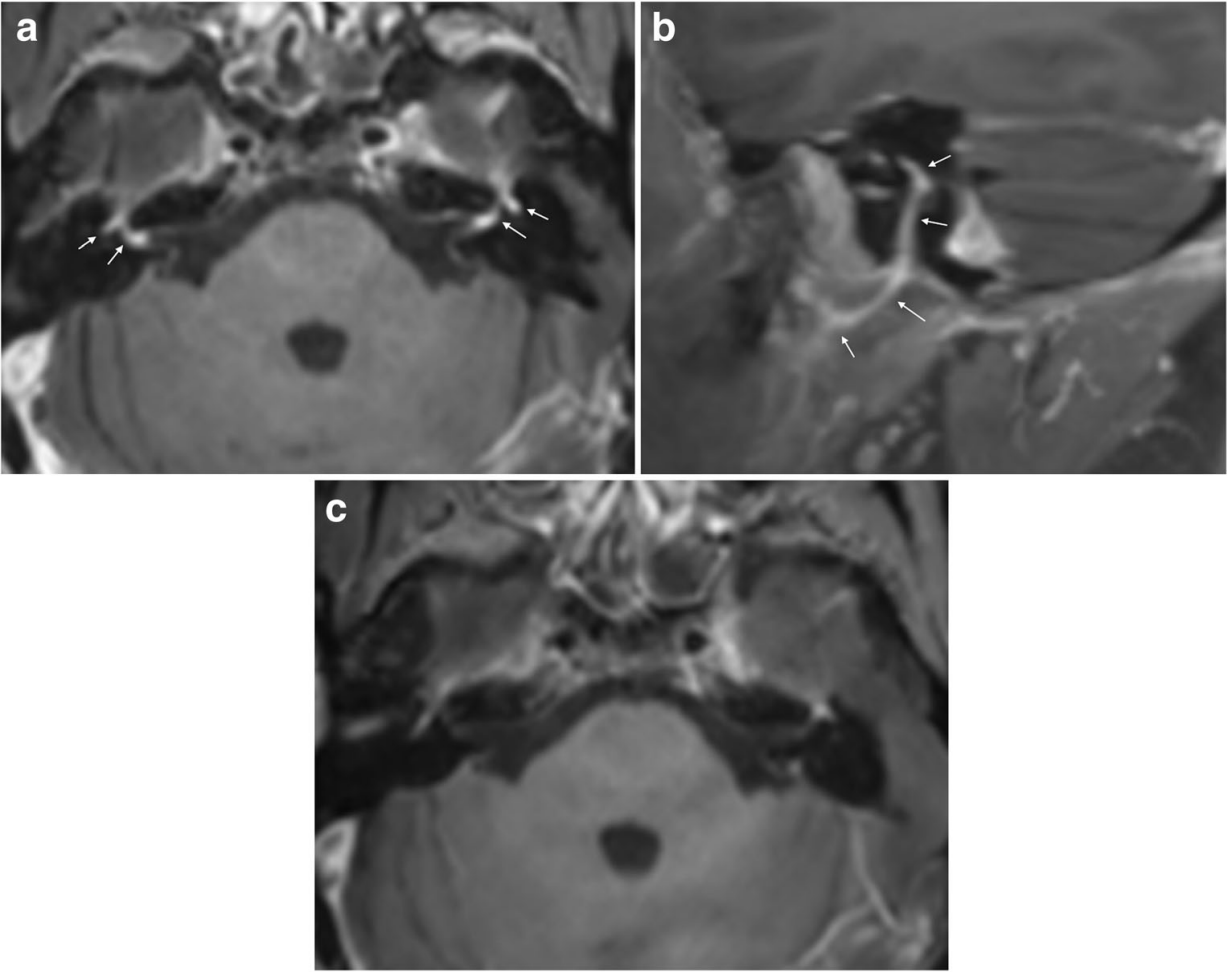
Facial neuritis in LNB with typical disease course in a 55-year-old female. She suffered from back pain radiating to chest, arms, head and neck, especially intense at night time, and after 2 weeks she also developed bilateral facial palsy. CSF analysis showed 594 cells/mm^3^ (normal range 0-5/mm^3^) and production of *Bb* specific IgG and IgM antibodies. Axial post-contrast fat suppressed T1-weighted image (**a**) showed intense enhancement in the distal intracanalicular, labyrinthine and geniculate ganglion segments of both facial nerves (arrows point to these structures which are mentioned from medial to lateral). 3 mm sagittal reformat (**b**) on the right side demonstrated the enhancement involving the tympanic and mastoid segment, as well as the parotid segment with the temporofacial and cervicofacial branches at the pes anserinus included (arrows point to these structures which are mentioned from cranial to caudal). Axial post-contrast fat suppressed T1-weighted image showed no abnormal enhancement of facial nerves 6 months after the treatment (**c**)

About 80% of cranial nerve involvement in LNB affect facial nerves, bilaterally in 25% of the cases [4, 66]. In a recently published retrospective evaluation of 68 LNB patients, facial palsy was the cause for hospitalisation in 50% of the patients [8]. A study by Ogrinc et al. of 77 patients with early LNB reported facial palsy in 36% [7]. MRI can demonstrate diffuse enhancement of cranial nerves, as shown in Fig. 2. There are no reports of nodular enhancement. Imaging evaluation of inflammation in the facial nerve can be challenging due to the lack of specificity concerning aetiology, in addition to normal enhancement seen in long segments of this nerve [67]. In the clinical setting, attention should be paid to asymmetry or marked intensity of enhancement in the geniculate ganglion, tympanic or mastoid segments, while there is normally no enhancement in the cisternal, intracanalicular, labyrinthine or parotid segments. To our knowledge, there are no data of the diagnostic accuracy of MRI in LNB facial neuritis. In 36 patients with idiopathic facial palsy a high sensitivity (100%) was found in contrast enhanced 3D T1 gradient echo sequence with fat suppression, and specificity was improved from 57 to 86% with the use of a contrast enhanced 3D FLAIR sequence [68]. In the workup of facial palsy, MRI can exclude a compressing lesion, brainstem infarct, perineural tumour spread or focal lesion such as schwannoma [67]. Certain concomitant MRI findings can be suggestive of some inflammatory etiologies such as Guillain-Barré syndrome, multiple sclerosis and sarcoidosis. In most cases of facial palsy, CSF analysis is an important supplemental test that will help to differentiate, and possibly identify, inflammatory and infectious etiologies.

## Myelitis

**Fig. 3:**
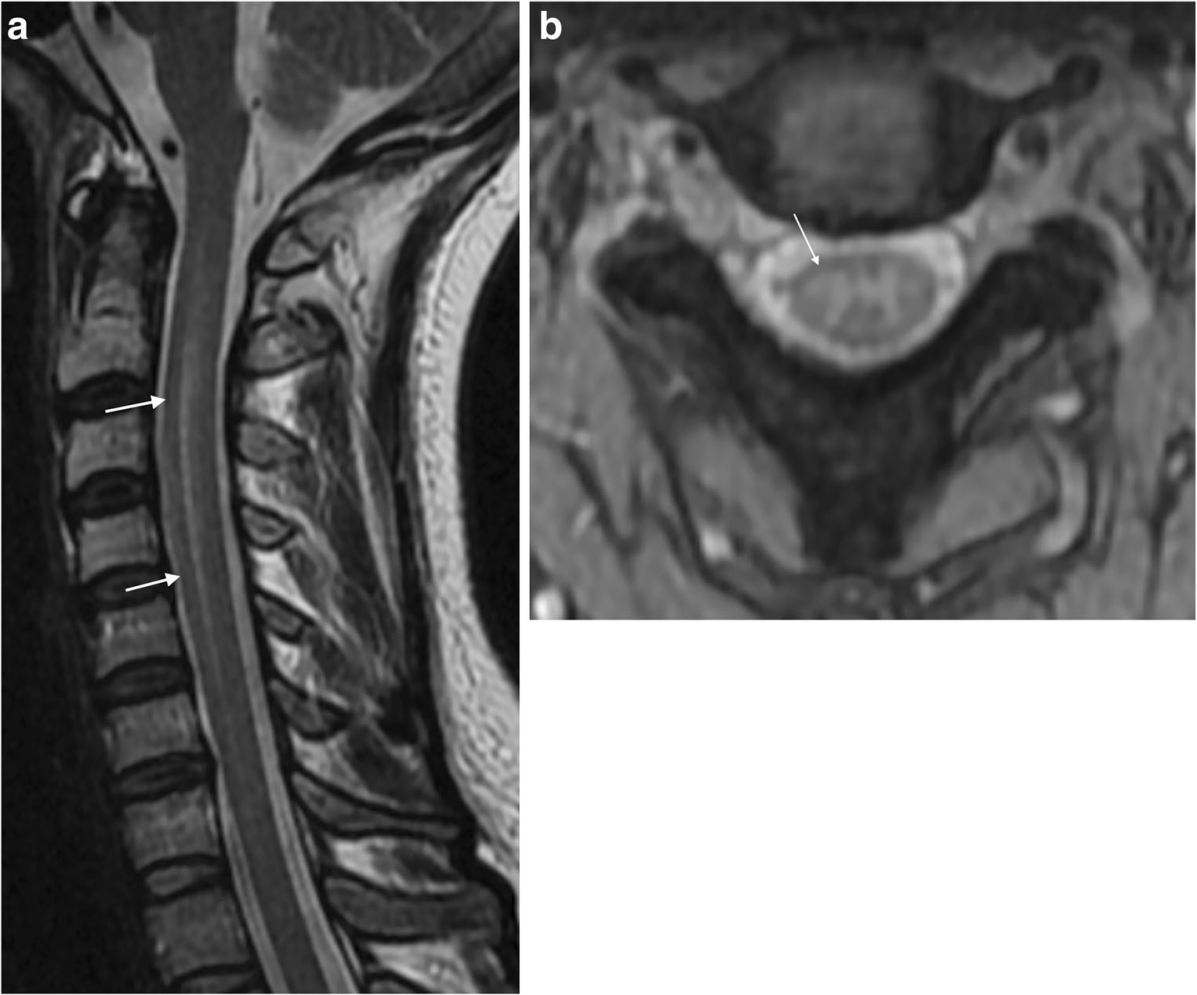
LNB myelitis in a 35-year old female who presented with pain radiating in the right arm, neck stiffness and general symptoms of headache, fatigue, nausea and vomiting. T2 weighted images showed subtle spinal cord hyperintensity, on sagittal image (**a**) from C2-C5 centrally located (arrows), and on axial T2 gradient echo image at the C2/C3 level (**b**), dominant affection adjacent to the right anterior horn was seen (arrow). CSF analysis showed increased cell count as well as intrathecal production of *Bb* specific IgM and IgG antibodies. Two months after treatment she had fully recovered. Seven years has passed since her symptom debut, and there has been no further incidence of neurological affection

Myelitis confirmed by MRI constituted 7% of the LNB manifestations leading to hospitalisation in the aforementioned study by Schwenkenbecher et al. [8]. In addition to the five cases from that study, we have found 11 adult case reports of LNB myelitis with abnormal MRI findings of the spinal cord substance [29, 34, 42, 69–76]. In all of them the cervical spinal cord was affected. The majority were, like the case presented in Fig. 3, longitudinally extensive and centrally or slightly anteriorly located. One case had a focal lesion at the C5 level [69], and another multifocal cervical lesions [75]. Enhancement patterns ranged from no enhancement, to nodular or diffusely extensive contrast enhancement. Also, one case report of a cervical spinal cord syrinx exists [73]. Lumbosacral myelitis and normal MRI findings have also been reported [39, 77]. In the setting of an acute transverse myelitis, additional findings of nerve root or leptomeningeal enhancement can be helpful imaging features in differentiating LNB from MS and other causes of centrally located myelitis such as acute disseminated encephalomyelitis and neuromyelitis optica spectrum disorders. Many infectious myelopathies can have combined manifestations with meningitis, radiculitis and encephalitis, among these are varicella-zoster virus, Epstein-Barr virus, tick-borne encephalitis (TBE) virus and cytomegalovirus [78]. History of pain can also be an important clue to the LNB diagnosis, but again CSF analysis is the key supplemental test.

## Peripheral neuropathy

**Fig. 4:**
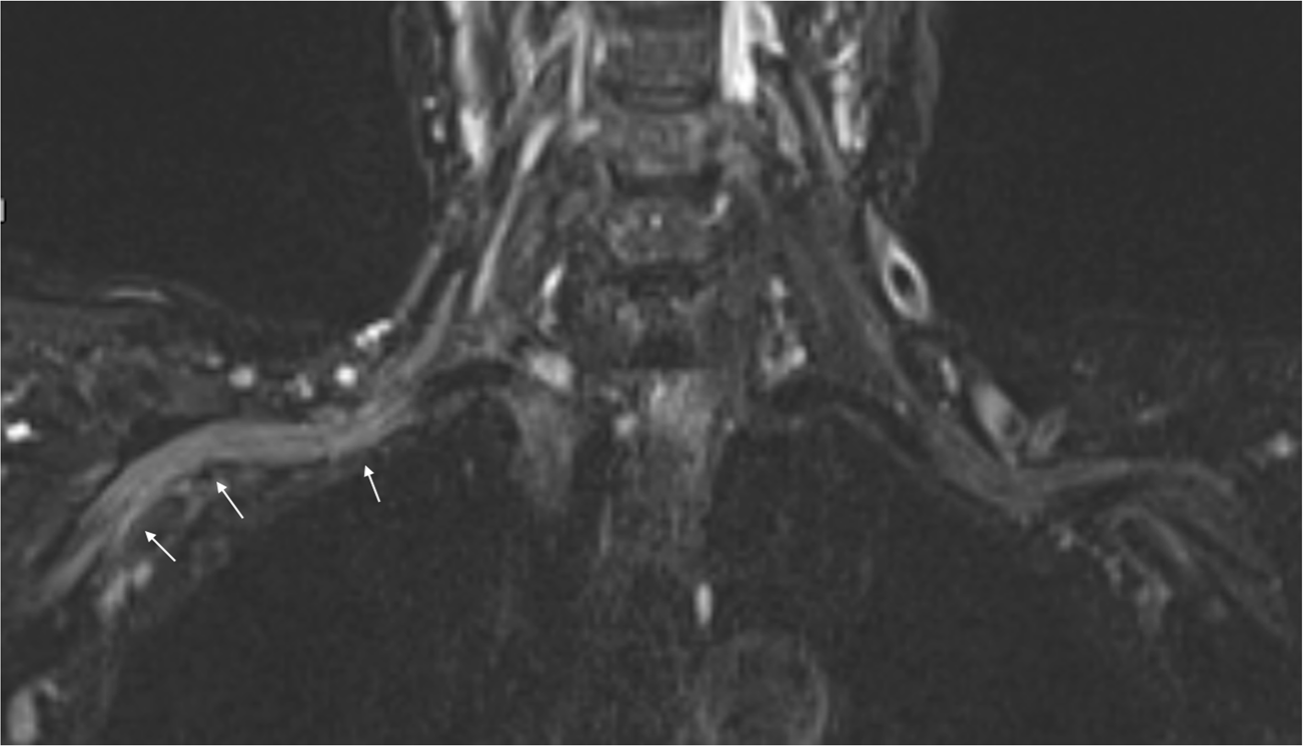
Brachial plexus neuritis in a 74-year-old woman with acute distal right arm paresis. In the preceding weeks she had nausea, vomiting and weight loss, and felt increasingly forgetful and unsteady. A fluid-sensitive coronal STIR sequence showed diffusely increased signal of the nerve bundle on the right side (arrows) compared to the left side. Lumbar puncture followed, and showed cell count of 101/mm^3^ as well as a positive *Bb* IgG antibody index (AI)

Bannwarth syndrome with radicular pain and/or facial palsy are typical LNB manifestations. Plexus neuritis or mononeuritis multiplex are other peripheral manifestations, seen in 5-10% of LNB cases [10]. This can present as a chronic asymmetric neuropathy, and will in such cases usually not have meningitis or intrathecal antibody production [79]. In neuritis due to LNB, nonspecific findings such as increased signal of nerve structures on fluid-sensitive sequence can be encountered. Figure 4 shows a case of acute peripheral motor nerve affection due to LNB brachial plexus neuritis. No symptoms of radiculitis preceded the motor affection in our patient, which is untypical [29, 80]. Due to the focal or regional distribution of symptoms in such patients, MRI will commonly supplement clinical, blood and electrodiagnostic tests, in case nerve entrapment or a mass lesion can be revealed [81].

## Encephalitis

**Fig. 5:**
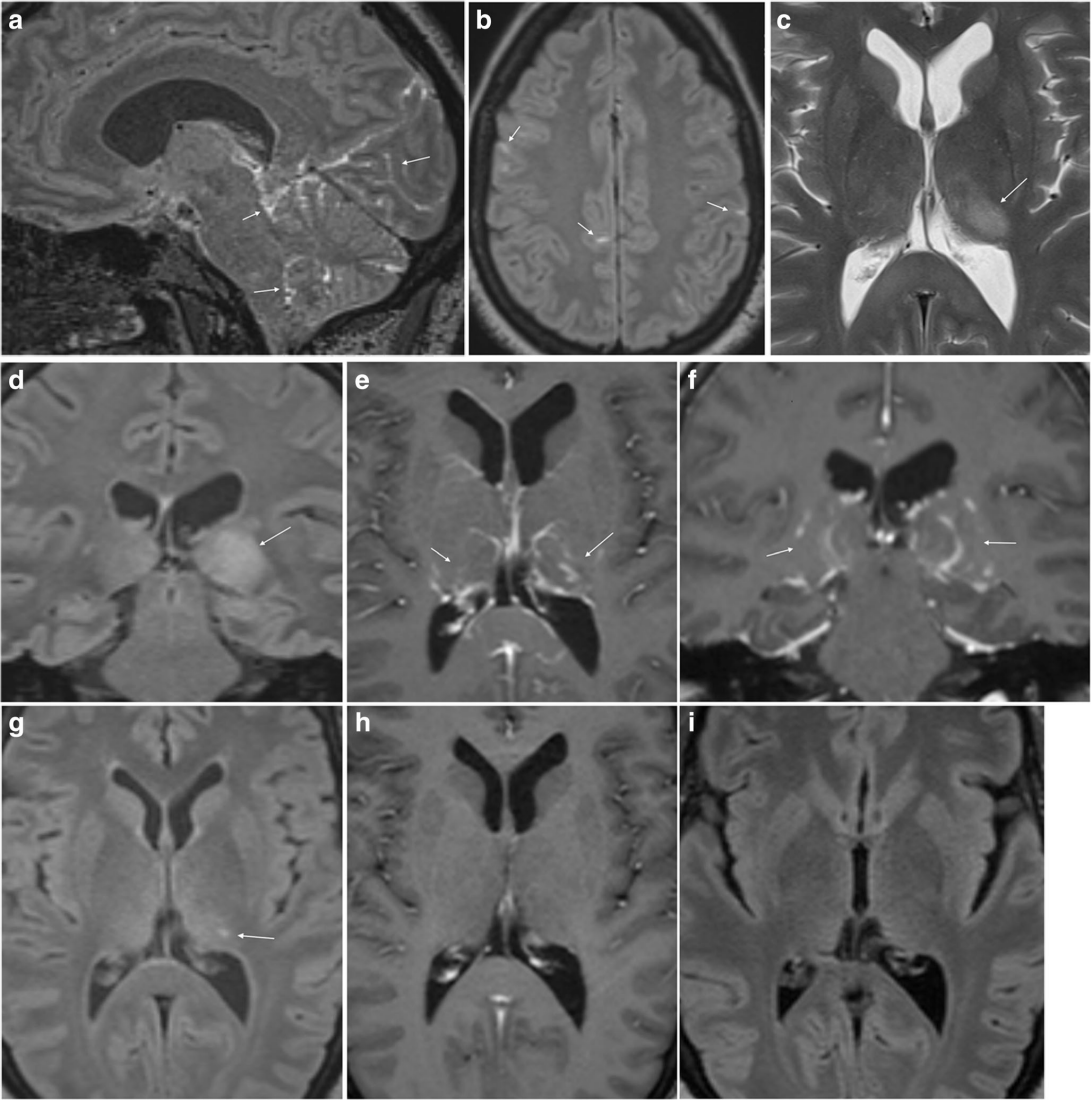
LNB meningoencephalitis in a 45-year-old female who presented with fatigue, headache, dizziness and vomiting. Neurological findings were tremor and unsteady gait. The CSF cell count was 270 cells/mm^3^ and there was a positive *Bb* IgG AI. Post-contrast sagittal (**a**) and axial (**b**) FLAIR images demonstrated patchy leptomeningeal enhancement over the cerebral convexities and in the posterior fossa (arrows). There was a hyperintense lesion in the left thalamus (arrow) on axial T2-weighted image (**c**) and coronal FLAIR image (**d**). There was no abnormality in the lesion on diffusion weighted images (not shown). Post-contrast axial (**e**) and coronal (**f**) T1-weighted images showed punctate and curvilinear uptake (arrows) in the thalamic lesion. Similar, subtle changes were present in the right thalamus. After 2 months there was only slight residual hyperintensity in the left thalamus (arrow) on axial FLAIR image (**g**) and no sign of enhancement on post-contrast axial T1 weighted image (**h**). One year after treatment there were no imaging abnormalities (**i**)

**Fig. 6:**
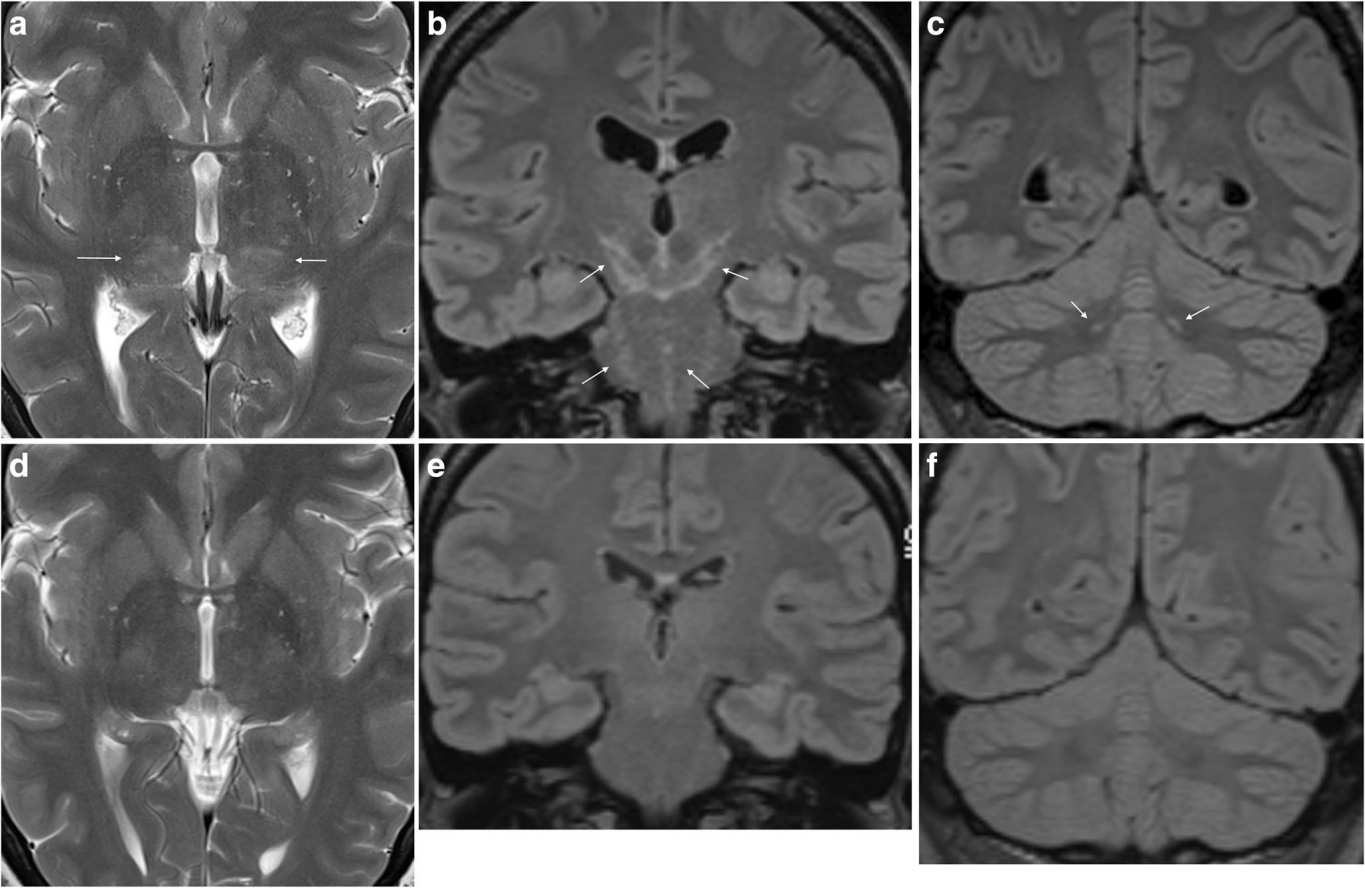
LNB encephalitis in a 40-year-old woman. She presented with 1 day history of headache, vomiting, dizziness and personality change. The CSF cell count was 182 cells/mm^3^. MRI showed symmetric diffuse and patchy T2 (**a**) and FLAIR (**b**, **c**) hyperintensity of inferior thalami (arrows in **a**), cerebral peduncles, mesencephalon, pons (arrows in **b**) and cerebellar dentate nuclei (arrows in **c**). There were no abnormalities on diffusion-weighted or post-contrast images (not shown). Further clinical and laboratory investigations revealed erythema migrans on the right thigh and a positive CSF *Bb* IgG AI. Tests for TBE antibodies were negative. She received antimicrobial treatment, and repeated MRI 8 weeks after the initial scan was normal (**d**–**f**)

Among the 68 patients studied by Schwenkenbecher et al., one adult and two children were clinically classified as acute encephalitis, and one of them had abnormal MRI findings [8]. Non-specific MRI findings of diffuse involvement of brain parenchyma are among the few case reports mentioned earlier, and has also occasionally been encountered in our institutions (Figs. 5 and 6). The case in Fig. 5 has a resemblance to CLIPPERS (chronic lymphocytic inflammation with pontine perivascular enhancement responsive to steroids), an entity thought to represent immune-mediated inflammation [82]. Interestingly, in our patient the abnormality resolved with antibiotic treatment only. Massengo et al. reported a case with similar type of punctate and linear enhancing lesions in cerebral and cerebellar parenchyma which responded to steroid treatment [32]. No specific pattern of involvement for LNB encephalitis can be identified from the few cases that exist. Still, of note is that it seems closer to the involvement pattern seen in rhombencephalitis, which also can present in combination with leptomeningeal enhancement in infections such as Listeria monocytogenes and tuberculosis. Involvement of insula, temporal lobe and limbic system, which is typical for herpes simplex virus infection, has not been reported in LNB [83]. We have seen cases with involvement of thalami, and this was also reported in a case of LNB encephalitis by Haene et al. [30]. Among other conditions with acute and subacute thalamic affection are Japanese encephalitis, acute disseminated encephalomyelitis, neuro-Beςhet, venous and arterial infarctions. Involvement of thalami, basal ganglia and cerebellum have also been proposed to be suggestive of viral tick-borne encephalitis [84, 85]. Tests for specific antibodies to both *Bb* and TBE virus should be included in the workup of patients with suspected encephalitis who live in or have travelled to areas where these tick-borne infections are endemic.

## Cerebral vasculitis

**Fig. 7:**
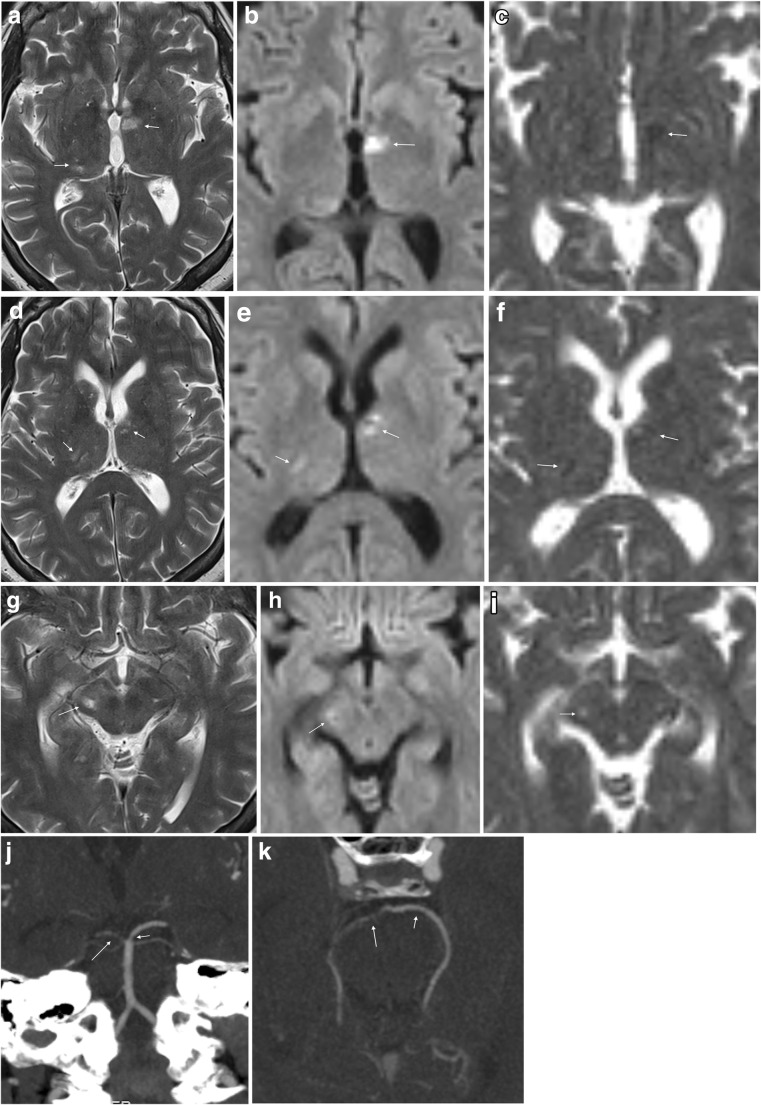
LNB vasculitis in a 55-year-old woman with strange behaviour for 3 days preceding hospital admission. She had incoherent speech and was disorientated. CSF cell count was 213 cells/mm^3^ and *Bb* IgG AI was positive. MRI revealed ischemic lesions of different ages in thalamus bilaterally (**a**–**f**), right cerebral peduncle (**g–i**) and right occipital cortex (not shown). Axial T2-weighted images (**a**, **d**, **g**), diffusion weighted B1000 images (**b**, **e**, **h**) and ADC maps (**c**, **f**, **i**) demonstrated the lesions. In contrast to the hypointense lesions on ADC maps in the thalami (arrows in c and f) consistent with cytotoxic edema in recent infarcts, the lesion in the right cerebral peduncle was hyperintense on both B1000 image (arrow in h) and ADC map (arrow in i), consistent with vasogenic edema and a more advanced stage of ischemic lesion. 5 mm MIP coronal (**j**) and axial (**k**) reformats of CT angiography examination demonstrated several short stenotic segments of both posterior cereberal arteries (arrows), most pronounced on the right side

LNB vasculitis is presumed to be caused by the inflammatory response to the spirochetal infection [16]. A German study calculated a 0.3% chance of cerebral vasculitis in Lyme borreliosis, and there may be an affinity for involvement of the posterior cerebral circulation in LNB vasculitis [44, 86]. Imaging is necessary to recognise this type of complication (Fig. 7). Diffusion weighted imaging can identify the restricted diffusion typical for acute and subacute ischemic lesions, a finding which has not been reported or seen in our experience in LNB encephalitic lesions. Angiographic examinations can demonstrate irregularities of the vessel lumen with occlusion or segmental narrowing or dilatation. Such findings support a diagnosis of vasculitis, and can be attributed to LNB in case of fulfilled diagnostic criteria as described previously. It is important for radiologists and clinicians to be aware of LNB as a possible cause for stroke and vasculitis in patients exposed to ticks.

## Conclusion

Non-specific imaging findings are a constant challenge to radiologists. Insight into clinical disease courses and laboratory test methods will aid in the multidisciplinary interaction that often is imperative to achieve an efficient patient workup and arrive at a correct diagnosis. This is especially true in LNB, where a wide spectrum of neurological, and sometimes general and cognitive symptoms can be encountered, as well as diverse imaging pathology from neuritis to meningitis, encephalitis and vasculitis. The primary role of imaging is to look for other causes to explain the clinical symptoms. This article can educate those engaged in imaging of the nervous system and serve as a comprehensive tool when LNB is in question.

## Abbreviations

AAN: American Academy of Neurology
ADC: Apparent diffusion coefficient
AI: Antibody index
Bb: Borrelia burgdorferi
CLIPPERS: Cenhancement responsive to steroids
CNS: Central nervous system
CSF: Cerebrospinal fluid
EFNS: European Federation of Neurological Societies
FLAIR: Fluid attenuated inversion recovery
LNB: Lyme neuroborreliosis
MRI: Magnetic resonance imaging
MS: Multiple sclerosis
PET: Positron emission tomography
SPECT: Single photon emission computed tomography
TBE: Tick-borne encephalitis
